# Gross anatomical features of the insular cortex in schizophrenia and schizotypal personality disorder: Potential relationships with vulnerability, illness stages, and clinical subtypes

**DOI:** 10.3389/fpsyt.2022.1050712

**Published:** 2022-11-18

**Authors:** Tsutomu Takahashi, Daiki Sasabayashi, Yoichiro Takayanagi, Atsushi Furuichi, Haruko Kobayashi, Yusuke Yuasa, Kyo Noguchi, Michio Suzuki

**Affiliations:** ^1^Department of Neuropsychiatry, University of Toyama Graduate School of Medicine and Pharmaceutical Sciences, Toyama, Japan; ^2^Research Center for Idling Brain Science, University of Toyama, Toyama, Japan; ^3^Arisawabashi Hospital, Toyama, Japan; ^4^Department of Radiology, University of Toyama Graduate School of Medicine and Pharmaceutical Sciences, Toyama, Japan

**Keywords:** magnetic resonance imaging, schizotypal, deficit schizophrenia, insula, gyrification, early neurodevelopment

## Abstract

**Introduction:**

Patients with schizophrenia have a higher number of insular gyri; however, it currently remains unclear whether the brain characteristics of patients with schizotypal personality disorder (SPD), a mild form of schizophrenia, are similar. It is also unknown whether insular gross anatomical features are associated with the illness stages and clinical subtypes of schizophrenia.

**Materials and methods:**

This magnetic resonance imaging study examined gross anatomical variations in the insular cortex of 133 patients with schizophrenia, 47 with SPD, and 88 healthy controls. The relationships between the insular gross anatomy and schizophrenia subgroups (71 first-episode and 58 chronic groups, 38 deficit and 37 non-deficit subtype groups) were also investigated.

**Results:**

The number of insular gyri was higher in the schizophrenia and SPD patients than in the controls, where the patients were characterized by well-developed accessory, middle short, and posterior long insular gyri. The insular gross anatomy did not significantly differ between the first-episode and chronic schizophrenia subgroups; however, the relationship between the developed accessory gyrus and more severe positive symptoms was specific to the first-episode group. The prevalence of a right middle short gyrus was higher in the deficit schizophrenia group than in the non-deficit group.

**Discussion:**

These findings suggest that schizophrenia and SPD patients may share an altered insular gross morphology as a vulnerability factor associated with early neurodevelopmental anomalies, which may also contribute to positive symptomatology in the early illness stages and clinical subtypes of schizophrenia.

## Introduction

The insular cortex is involved in a range of cognitive functions as a “limbic integration cortex” (1) and is characterized by large inter-individual variations in the gross gyral organization (2, 3). The anterior subdivision (short insular cortex) is typically composed of an accessory and three principal short gyri (anterior, middle, and posterior), while the accessory gyrus (AG) and middle short gyrus (MSG) are frequently underdeveloped or absent (up to 50–70%) in general population (4–6). The posterior subdivision (long insular cortex) consists of the anterior and posterior long insular gyri, where the posterior long gyrus (PLG) is missing in between 10 and 20% of human brains (4–6). The significance of the effects of these anatomical variations on the function of the insular cortex has not yet been established; however, we recently reported a higher number of insular gyri with a well-developed AG, MSG, and PLG in patients with first-episode schizophrenia than in controls (7). Since gross brain folding patterns do not markedly change after birth (8), this finding in schizophrenia potentially reflects anomalous neurodevelopment during the mid to late fetal period, during which insular cortical folds are formed (9, 10). Nevertheless, there is currently no evidence to show similar features of the insular gross anatomy in patients with schizophrenia spectrum disorders, who may share early neurodevelopmental pathologies associated with vulnerability to psychosis (11).

Schizotypal personality disorder (SPD) (12), or schizotypal disorder (13), is a milder form within the schizophrenia spectrum and is characterized by attenuated forms of schizophrenic features without overt psychosis. SPD patients are considered to share biological similarities with patients with full-blown schizophrenia, potentially reflecting a common vulnerability (11, 14). Schizophrenia spectrum disorders partly share brain abnormalities, such as diverse cortical hyper-gyrification (15, 16), which may reflect deviations in early neurodevelopment (17, 18). On the other hand, gray matter reductions in the insular cortex appear to be specific to schizophrenia among schizophrenia spectrum disorders (19–23). To the best of our knowledge, magnetic resonance imaging (MRI) studies have not yet specifically examined variations in the insular gross anatomy in SPD patients.

We previously demonstrated that the gross anatomical features of the insular cortex correlated with positive symptomatology in first-episode schizophrenia (7); however, their potential contribution to clinical characteristics at later illness stages and clinical subtypes was not examined. Patients with the deficit subtype of schizophrenia, who are found in approximately 15% of first-episode and 25–30% of more chronic patients, have a trait-like feature of primary and persistent negative symptoms even during remission periods (24, 25). Unlike the DSM/ICD subtypes of schizophrenia (12, 13) based on symptom profiles (e.g., paranoid, disorganized, and undifferentiated), the deficit/non-deficit categorization is highly stable over time and the patients with deficit subtype have rather homogeneous poor outcome as demonstrated in longitudinal clinical follow-up (25). Previous MRI studies on deficit schizophrenia revealed that the patients with this specific subtype may exhibit prominent abnormalities in neurodevelopment (26, 27), as suggested by gross brain changes, including an altered surface morphology in the fronto-temporal regions (28, 29). To obtain a more detailed understanding of the role of insular gross anatomical features in the pathophysiology of schizophrenia, further studies in different illness stages and in specific clinical subtypes, particularly those with a prominent neurodevelopmental pathology (i.e., deficit schizophrenia), are warranted.

We herein used MRI to examine the insular gross anatomy of SPD patients and patients with schizophrenia of different illness stages (first-episode and chronic) and subtypes (deficit and non-deficit). Due to shared neurodevelopmental pathologies in the schizophrenia spectrum and prominent neurodevelopmental abnormalities in deficit schizophrenia as described above, we expected SPD patients to have an elevated number of insular gyri, similar to schizophrenia, and this change to be prominent in the deficit schizophrenia subgroup. We also investigated whether the insular gross anatomy affects clinical characteristics even in the chronic stages of schizophrenia.

## Materials and methods

### Participants

Participants in the present study comprised 133 patients with schizophrenia, 47 with SPD, and 88 healthy controls (Table 1). Their physical condition was good at the time of MRI and they had no previous history of serious illnesses requiring medical treatment (e.g., hypertension, seizure, head injury, diabetes, and thyroid diseases), oral steroid use, or substance use disorders. Among 268 participants, the insular gross anatomy of 66 first-episode schizophrenia patients and 66 healthy controls was reported elsewhere (7). This study aimed to examine the insular anatomy in our SPD sample as well as in an expanded schizophrenia sample with different illness duration (i.e., first-episode vs. chronic patients) and clinical characteristics (i.e., deficit *vs.* non-deficit subtypes) to explore the role of vulnerability to psychosis, illness stages, and subtypes. The recruitment strategies and inclusion criteria of participants were fully described in previous studies (16, 23, 30, 31).

**TABLE 1:** Sample characteristics and gross insular morphology of participants.

	Controls (*N* = 88)	SPD (*N* = 47)	Sz (*N* = 133)	Group differences
M/F	49/39	29/18	67/66	Chi-square = 1.93, *p* = 0.382
Age (years)	24.1 ± 6.0	25.0 ± 5.4	26.8 ± 6.3	*F*(2, 265) = 5.45, *p* = 0.005; Controls < Sz
Handedness (Right/left/mixed)	88/0/0	47/0/0	126/1/6	Fisher’s exact test, *p* = 0.095
Height (cm)	166.3 ± 7.8	165.9 ± 8.7	164.2 ± 7.9	*F*(2, 265) = 1.57, *p* = 0.209
Education (years)	15.7 ± 3.0	13.1 ± 2.0	13.5 ± 2.0	*F*(2, 265) = 27.22, *p* < 0.001; SPD, Sz < Controls
Parental education (years)^a^	13.0 ± 2.3	12.3 ± 1.7	12.5 ± 2.1	*F*(2, 255) = 1.75, *p* = 0.176
Onset age (years)	–	–	22.6 ± 5.4	–
Duration of illness (years)	–	–	4.0 ± 4.4	–
Medication dose (HPD equivalent, mg/day)	–	4.8 ± 5.7	9.9 ± 8.4	*F*(1, 178) = 15.09, *p* < 0.001; SPD < Sz
Duration of medication (years)	–	1.5 ± 3.0	2.8 ± 3.8	*F*(1, 178) = 4.20, *p* = 0.042; SPD < Sz
Medication type (Atypical/typical/mixed)	–	26/14/0	78/45/6	Fisher’s exact test, *p* = 0.503
Total SAPS scores^b^	–	16.0 ± 9.2	29.3 ± 22.8	*F*(1, 169) = 14.46, *p* < 0.001; SPD < Sz
Total SANS scores^b^	–	41.9 ± 21.7	50.5 ± 22.6	*F*(1, 169) = 4.90, *p* = 0.028; SPD < Sz
Number of short gyri				*F*(2, 262) = 38.89, *p* < 0.001; Controls < SPD, Sz
Left	2.78 ± 0.58	3.32 ± 0.66	3.35 ± 0.69	
Right	2.70 ± 0.59	3.36 ± 0.67	3.18 ± 0.61	
Number of long gyri				*F*(2, 262) = 9.56, *p* < 0.001; Controls < SPD, Sz in M
Left	1.85 ± 0.42	2.04 ± 0.20	2.01 ± 0.40	
Right	1.85 ± 0.44	1.98 ± 0.33	2.03 ± 0.37	
AG (Absent/underdeveloped/developed)				
Left	36/33/19	17/16/14	44/28/61	Chi-square = 15.79, *p* = 0.003
Right	44/32/12	13/15/19	56/36/41	Chi-square = 14.85, *p* = 0.005
MSG (Absent/underdeveloped/developed)				
Left	16/25/47	1/3/43	7/15/111	Chi-square = 34.28, *p* < 0.001
Right	11/31/46	2/3/42	9/17/107	Chi-square = 29.95, *p* < 0.001
PLG (Absent/present)				
Left	15/73	0/47	10/123	Chi-square = 11.55, *p* = 0.003
Right	16/72	3/44	9/124	Chi-square = 8.38, *p* = 0.015

Values represent means ± SDs unless otherwise stated. AG, accessory gyrus; ALG, anterior long gyrus; F, female; HPD, haloperidol; M, male; MSG, middle short gyrus; PLG, posterior long gyrus; SANS, scale for the assessment of negative symptoms; SAPS, scale for the assessment of positive symptoms; SPD, schizotypal personality disorder; Sz, schizophrenia.

^a^Data not available for one control, four SPD, and five Sz subjects.

^b^Data not available for two SPD and seven Sz patients.

Briefly, we enrolled schizophrenia and SPD patients at the Department of Neuropsychiatry, Toyama University Hospital and they were diagnosed by experienced psychiatrists based on a structured interview using the Comprehensive Assessment of Symptoms and History (32) and the Scale for the Assessment of Negative Symptoms and the Scale for the Assessment of Positive Symptoms (SANS/SAPS) (33). The schizophrenia group was also assessed using the Brief Psychiatric Rating Scale (BPRS) (34) for the purpose of clinical subgrouping.

Schizophrenia patients fulfilling the ICD-10 research criteria (13) were operationally categorized into first-episode [illness duration ≤ 1 year (*N* = 54) or under psychiatric hospitalization for the first time (*N* = 17)] and chronic [illness duration ≥ 3 years (*N* = 58)] subgroups (35, 36). As previously described in detail (28, 29, 37), we divided the patients into deficit and non-deficit schizophrenia subgroups based on scores for Proxy for the Deficit Syndrome (PDS) (38), which were obtained as follows using BPRS scores: blunted affect – (anxiety + guilt feelings + depressive mood + hostility items). To reduce false classification, patients with the top and bottom 25% of PDS scores among the whole schizophrenia sample were categorized into the deficit and non-deficit subgroups, respectively (39).

All schizotypal patients met the DSM Axis II diagnosis of SPD, with 13 having a history of transient quasi-psychotic episodes fulfilling the DSM Axis I diagnosis of brief psychotic disorder (12). They also fulfilled the ICD-10 research criteria of schizotypal disorder (13). None of these patients had developed schizophrenia during clinical follow-ups for at least 2 years. Table 1 shows the status of medication and clinical data on schizophrenia and SPD patients.

Following screening by a questionnaire on personal and family medical histories (40), healthy controls were enrolled from the community, hospital staff, and university students. None had a personal or family history of psychiatric illness among first-degree relatives. The Committee of Medical Ethics of the University of Toyama approved this study (ID: I2013006). In accordance with the Declaration of Helsinki, written informed consent was obtained from all participants after a full description of the study protocol. If a participant was younger than 20 years old, written consent was also obtained from a parent/guardian.

### MR image acquisition and processing

One-millimeter-thick T1-weighted images were obtained in the sagittal plane with the three-dimensional gradient-echo sequence FLASH (fast low-angle shots) using a 1.5T Magnetom Vision MR scanner (Siemens Medical System, Inc., Erlangen, Germany) under the following imaging conditions: TR = 24 ms, TE = 5 ms, flip angle = 40^°^, field of view = 256 mm, matrix = 256 × 256 pixels, and voxel size = 1 × 1 × 1 mm.

Using Dr. View software (Infocom, Tokyo, Japan), MR images were reconstructed into 1-mm-thick coronal images perpendicular to the inter-commissural line after three-dimensional tilt correction.

### Assessment of anatomical variations in the insula

**FIGURE 1:**
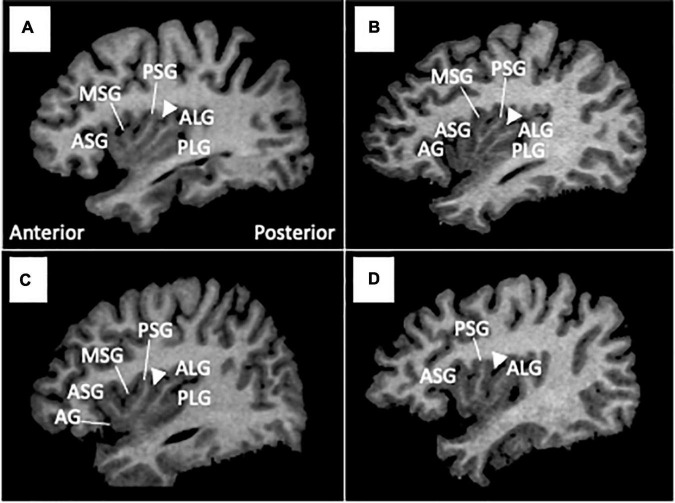
Insular gross anatomical variations in sample MR images in sagittal views. Coronal and axial views were simultaneously referred to in assessments of gyral development. Arrowheads indicate the location of the central insular sulcus, which subdivides the short (anterior) and long (posterior) insular cortices. The ASG, PSG, and ALG were well-developed in all participants in this study, while the AG and MSG were absent [subject **(D)**], underdeveloped [subject **(C)**], or well-developed [subject **(B)**]. The PLG was present in most subjects **(A–C)**, but was not observed in subject **(D)**. AG, accessory gyrus; ALG, anterior long gyrus; ASG, anterior short gyrus; MSG, middle short gyrus, PLS, posterior long gyrus; PSG, posterior short gyrus.

As described in detail elsewhere (7), one rater who was blinded to the identities of subjects assessed the insular gross anatomy primarily using the sagittal view (Figure 1). Briefly, the AG and MSG were classified as absent, underdeveloped (i.e., identifiable, but does not extend to the convex surface of the insula), or developed based on the criteria reported by Wysiadecki et al. (6). The PLG was classified as present or absent because it is developed in most hemispheres (> 85%) and hypoplasia is rarely observed (4, 6). The anterior short gyrus (ASG), posterior short gyrus (PSG), and anterior long gyrus (ALG) were well-developed in all participants in this study. Regarding the number of insular gyri, only well-developed gyri were counted.

The classification reliabilities of insular gyri were examined in a subset of 10 brains that were randomly selected (20 hemispheres); intra- (TT) and inter-rater (TT and DS) reliabilities were > 0.89 for the number (intraclass correlation coefficients) and development classification (Cronbach’s α).

### Statistical analysis

Group differences in demographic and clinical data were assessed by the χ^2^-test or an analysis of variance (ANOVA).

The developmental patterns of the AG, MSG, and PLG were exploratory compared between 3 groups (controls, SPD, and schizophrenia) using the χ^2^-test or Fisher’s exact test, where Benjamini-Hochberg procedure was used to decrease the false discovery rate. Lower-order comparisons (e.g., between two groups), which were not corrected for multiple comparisons due to prior hypothesis that both patient groups would similarly have well-developed insular gyri, were performed when significant group differences were found. The number of short (AG, ASG, MSG, and PSG) and long (ALG and PLG) gyri was log-transformed because of a skewed distribution (tested by Kolmogorov–Smirnov tests) and then compared between groups by ANOVA, with diagnosis and sex as between-subject factors and hemisphere as a within-subject variable.

Spearman’s correlation analysis with the Bonferroni correction was used to investigate relationships between the number of short insular gyri and clinical variables (age at disease onset in schizophrenia, the dose of medication and total SANS/SAPS scores in both patient groups). We did not use the durations of illness and medication here, because these variables were unlikely to be related to the insular gross anatomy, a stable brain characteristic. The first-episode and chronic schizophrenia groups were separately treated to explore the role of illness stages. Long gyri were not used in correlation analyses because there were two in most hemispheres (87.2%). The potential effects of insular gyral development on these clinical variables were examined by ANOVA, with the development pattern (developed *vs.* underdeveloped or absent) as a between-subject factor. Clinical variables were log-transformed, except for the total SAPS score in the schizotypal group and the SANS score, due to their non-normal distributions (Kolmogorov–Smirnov tests). *Post hoc* Scheffé’s tests were employed. The significance level was defined as *p* < 0.05.

## Results

### Demographic and clinical characteristics

No significant differences were observed in sex, handedness, or parental education between the groups; however, schizophrenia patients were older than healthy controls (Table 1). The education level was higher in healthy controls than in patient groups. The schizophrenia group was more symptomatic and received more medication than the SPD group (Table 1).

No significant differences were observed in age, handedness, personal or parental education, age at disease onset, illness duration, or medication (dose, type, and duration) between the deficit and non-deficit schizophrenia subgroups (27–29); however, a difference was noted in the sex ratio (Table 2). The deficit subgroup was characterized by a prominent blunted affect with less severe positive symptoms (Table 2).

**TABLE 2:** Sample characteristics and gross insular morphology of first-episode (FE) and chronic (C) schizophrenia.

	FE-Sz (*N* = 71)	C-Sz (*N* = 58)	Group differences
M/F	40/31	25/33	Chi-square = 2.24, *p* = 0.094
Age (Years)	24.3 ± 5.5	29.8 ± 6.3	*F*(1, 127) = 28.74, *p* < 0.001
Handedness (Right/left/mixed)	69/0/2	53/1/4	Fisher’s exact test, *p* = 0.304
Height (cm)	164.7 ± 7.8	163.9 ± 7.5	*F*(1, 127) = 0.35, *p* = 0.554
Education (Years)	13.5 ± 1.9	13.5 ± 2.0	*F*(1, 127) = 0.07, *p* = 0.785
Parental education (Years)^a^	12.9 ± 2.1	12.5 ± 2.1	*F*(1, 123) = 6.77, *p* = 0.010
Onset age (years)	23.3 ± 5.3	21.6 ± 5.3	*F*(1, 127) = 3.57, *p* = 0.061
Duration of illness (years)	0.9 ± 1.0	8.0 ± 3.9	*F*(1, 127) = 217.88, *p* < 0.001
Medication dose (HPD equivalent, mg/day)	10.2 ± 8.5	9.9 ± 8.4	*F*(1, 127) = 0.03, *p* = 0.867
Duration of medication (years)	0.6 ± 1.0	5.5 ± 4.3	*F*(1, 127) = 87.44, *p* < 0.001
Medication type (atypical/typical/mixed)	50/18/1	26/25/5	Fisher’s exact test, *p* = 0.005
Total SAPS scores^b^	27.3 ± 23.0	33.3 ± 22.2	*F*(1, 121) = 2.10, *p* = 0.150
Total SANS scores^b^	51.7 ± 24.8	48.9 ± 19.6	*F*(1, 121) = 0.46, *p* = 0.501
Number of short gyri			*F*(1, 125) = 1.67, *p* = 0.200
Left	3.43 ± 0.60	3.26 ± 0.76	
Right	3.24 ± 0.62	3.16 ± 0.59	
Number of long gyri			*F*(1, 125) = 0.88, *p* = 0.350
Left	1.97 ± 0.38	2.03 ± 0.42	
Right	2.01 ± 0.36	2.03 ± 0.37	
AG (Absent/underdeveloped/developed)			
Left	22/15/34	21/12/25	Chi-square = 0.42, *p* = 0.809
Right	29/19/23	24/16/18	Chi-square = 0.03, *p* = 0.986
MSG (Absent/underdeveloped/developed)			
Left	1/7/63	6/7/45	Fisher’s exact test, *p* = 0.060
Right	5/10/56	3/6/49	Fisher’s exact test, *p* = 0.736
PLG (Absent/present)			
Left	6/65	4/54	Fisher’s exact test, *p* = 1.000
Right	4/67	5/53	Fisher’s exact test, *p* = 0.730

Values represent means ± SDs unless otherwise stated. AG, accessory gyrus; ALG, anterior long gyrus; F, female; HPD, haloperidol; M, male; MSG, middle short gyrus; PLG, posterior long gyrus; SANS, scale for the assessment of negative symptoms; SAPS, scale for the assessment of positive symptoms; Sz, schizophrenia.

^a^Data not available for four Sz patients.

^b^Data not available for six Sz patients.

### Gross variations in insular gyri

**FIGURE 2:**
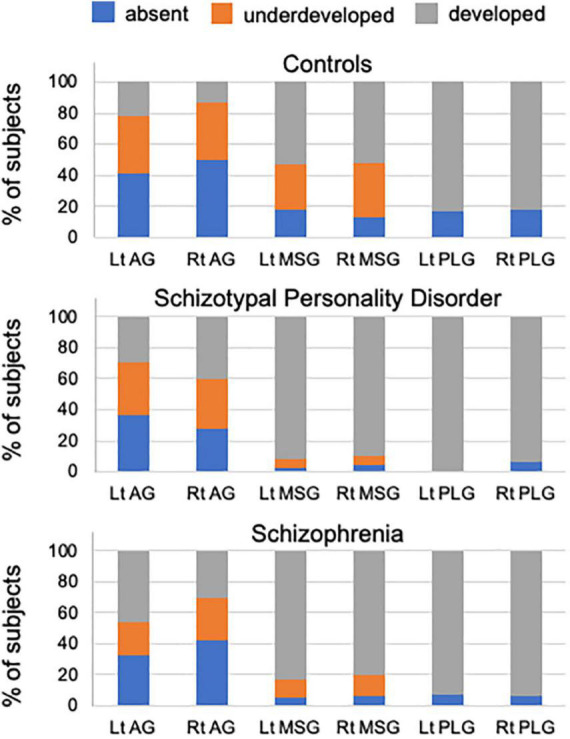
Percentage of insular gyral development in healthy controls, schizotypal patients, and schizophrenia patients. AG, accessory gyrus; MSG, middle short gyrus; PLG, posterior long gyrus.

The degree of gyral development for the AG, MSG, and PLG significantly differed between healthy controls and patient groups (i.e., schizophrenia and SPD groups), but not between patient groups (Table 1 and Figure 2). The AG (left, χ^2^ = 13.51, *p* < 0.001; right, χ^2^ = 8.58, *p* = 0.003), MSG (left, χ^2^ = 19.99, *p* < 0.001; right, χ^2^ = 19.74, *p* < 0.001), and PLG (left, χ^2^ = 4.79, *p* = 0.029; right, χ^2^ = 6.88, *p* = 0.009) were significantly more well-developed bilaterally in schizophrenia patients than in healthy controls. The right AG (χ^2^ = 12.43, *p* < 0.001), bilateral MSG (left, Fisher’s exact test, *p* < 0.001; right, χ^2^ = 18.57, *p* < 0.001), and left PLG (Fisher’s exact test, *p* = 0.001) were significantly more well-developed in the SPD group than in healthy controls. Among schizophrenia patients, the right MSG was more developed in males than in females (χ^2^ = 4.97, *p* = 0.026), while no other significant effects were noted involving sex and hemisphere for the degree of insular gyral development.

The number of short gyri was higher in the schizophrenia and SPD groups (Scheffé’s test, *p* < 0.001) than in healthy controls (Table 1). A significant group-by-sex interaction [*F*(2, 262) = 6.36, *p* = 0.002] was observed for long gyri, with male schizophrenia (Scheffé’s test, *p* = 0.005) and SPD (Scheffé’s test, *p* = 0.001) patients having a higher number than healthy male controls. These results remained the same even when age and medication (dose/duration) were used as covariates.

No significant differences were noted in the number or development patterns of insular gyri between the first-episode and chronic subgroups (Table 2). Because the results of a largely overlapping (*N* = 66/71) first-episode schizophrenia cohort have been reported elsewhere (7), we also demonstrate the results excluding the first-episode schizophrenia patients as Supplementary Table 1; the results remained essentially the same as the original results using whole schizophrenia sample (*N* = 133).

No significant differences were observed in the number of insular gyri between the deficit and non-deficit schizophrenia subgroups, whereas the prevalence of a well-developed right MSG was higher in the deficit subgroup than in the non-deficit subgroup (χ^2^ = 4.79, *p* = 0.029) (Table 3).

**TABLE 3:** Sample characteristics and gross insular morphology of deficit and non-deficit subtypes of schizophrenia.

	D-Sz (*N* = 38)	ND-Sz (*N* = 37)	Group differences
M/F	22/16	12/25	Chi-square = 4.90, *p* = 0.027
Age (Years)	27.1 ± 6.2	27.1 ± 7.5	*F*(1, 73) < 0.01, *p* = 0.984
Total BPRS score	36.5 ± 9.5	49.5 ± 12.0	*F*(1, 73) = 27.45, *p* < 0.001; D-Sz < ND-Sz
PDS score	–1.8 ± 1.4	–10.1 ± 1.8	*F*(1, 73) = 504.04, *p* < 0.001; ND-Sz < D-Sz
SAPS			Group-by-subscore interaction; *F*(3, 219) = 12.58, *p* < 0.001
Hallucinations	5.3 ± 7.5	13.5 ± 8.2	*Post hoc* comparison, *p* < 0.001; D-Sz < ND-Sz
Delusions	8.1 ± 8.0	19.0 ± 9.6	*Post hoc* comparison, *p* < 0.001; D-Sz < ND-Sz
Bizarre behavior	4.5 ± 4.0	5.5 ± 4.4	–
Positive formal thought disorder	3.7 ± 5.6	6.8 ± 8.9	–
SANS			Group-by-subscore interaction; *F*(4, 292) = 5.25, *p* < 0.001
Blunted affect	16.0 ± 8.8	12.7 ± 10.3	*Post hoc* comparison, *p* = 0.004; ND-Sz < D-Sz
Alogia	8.0 ± 5.6	6.9 ± 4.3	–
Avolition-apathy	10.8 ± 5.0	10.7 ± 4.8	–
Anhedonia-asociality	10.6 ± 5.8	12.8 ± 7.9	–
Attention deficit	7.5 ± 4.7	10.4 ± 4.1	*Post hoc* comparison, *p* = 0.011; D-Sz < ND-Sz
Number of short gyri			*F*(1, 71) = 2.31, *p* = 0.133
Left	3.39 ± 0.68	3.32 ± 0.71	
Right	3.26 ± 0.55	3.16 ± 0.60	
Number of long gyri			*F*(1, 71) = 1.68, *p* = 0.200
Left	2.05 ± 0.40	1.95 ± 0.33	
Right	1.97 ± 0.28	2.03 ± 0.29	
AG (absent/underdeveloped/developed)			
Left	12/8/18	10/10/17	Chi-square = 0.42, *p* = 0.811
Right	18/10/10	14/8/15	Chi-square = 1.71, *p* = 0.425
MSG (absent/underdeveloped/developed)			
Left	2/3/33	2/5/30	Fisher’s exact test, *p* = 0.799
Right	2/1/35	2/8/27	Fisher’s exact test, *p* = 0.028
PLG (absent/present)			
Left	2/36	3/34	Fisher’s exact test, *p* = 0.674
Right	3/35	1/36	Fisher’s exact test, *p* = 0.615

Values represent means ± SDs unless otherwise stated.

AG, accessory gyrus; ALG, anterior long gyrus; BPRS, Brief Psychiatric Rating Scale; D-Sz, deficit schizophrenia; F, female; M, male; MSG, middle short gyrus; ND-Sz, non-deficit schizophrenia; PDS, Proxy for the Deficit Syndrome; PLG, posterior long gyrus; SANS, scale for the assessment of negative symptoms; SAPS, scale for the assessment of positive symptoms.

### Relationships between the insular anatomy and clinical variables

A higher number of left short gyri was associated with a younger onset age (*rho* = –0.366, *p* = 0.002) and higher medication dose (*rho* = 0.288, *p* = 0.015) in first-episode schizophrenia patients, but not in chronic schizophrenia patients. The number of right short gyri in SPD patients was also related to a higher SANS score (*rho* = 0.214, *p* = 0.013). Among these results, the relationship with onset age in first-episode schizophrenia patients remained after the Bonferroni correction for multiple comparisons [22 comparisons, *p* < 0.0023 (0.05/22)].

Schizophrenia patients with a left developed AG had a younger onset age than those without in the first-episode subgroup [*F*(1, 69) = 9.47, *p* = 0.003], but not in the chronic subgroup. Similarly, schizophrenia patients with a right developed AG had a higher SAPS score than those without only for the first-episode subgroup [*F*(1, 67) = 4.43, *p* = 0.039]. A developed right AG was also related to a higher SANS score in the SPD group [*F*(1, 43) = 4.11, *p* = 0.049].

## Discussion

To the best of our knowledge, this is the first MRI study to demonstrate that patients with established schizophrenia (both first-episode and chronic stages) and SPD had a higher number of short and long insular gyri than healthy controls, potentially representing a common neurodevelopmental pathology within the schizophrenia spectrum. We also showed that a well-developed AG in schizophrenia was associated with an earlier onset and severe positive symptoms in first-episode, but not chronic patients. Furthermore, a well-developed MSG in schizophrenia was related to a subgroup with primary and persistent negative symptoms (i.e., deficit schizophrenia). Therefore, the gross anatomy of the insular cortex appears to contribute to vulnerability to psychosis, clinical features in early illness stages, and the clinical subtype of schizophrenia.

The present results showing an increased number of insular gyri in SPD, similar to schizophrenia, is considered to reflect common insults in the process of fetal insular gyration that predominantly occur between 17 and 35 weeks of gestation (9, 10). Previous MRI studies on shared abnormalities in early neurodevelopmental markers, such as the small adhesio interthalamica (41), an altered surface morphology in the orbitofrontal region (42, 43), and diverse cortical hyper-gyrification (16), support common neurodevelopmental pathologies among schizophrenia spectrum disorders (11, 14). Since aberrant neurodevelopmental processes associated with gyral formation *in uteri* may lead to neural dysconnectivity (17, 18), our results showing gross insular changes and their contribution to negative symptoms in SPD patients are partly consistent with the diffusion tensor imaging findings of schizotypal subjects with altered connectivity involving the insular cortex, which is associated with clinical symptoms and cognitive impairments (44, 45). The insular gray matter volume, which exhibits a progressive decline in the early stages of schizophrenia (20, 21), is preserved in SPD (19, 23); therefore, the results obtained in the present study appear to support the insular morphology in schizophrenia spectrum disorders having multiple pathological processes. Gross anatomical features may represent a vulnerability to psychosis that is attributable to prenatal neurodevelopment, while the gray matter volume more reflects dynamic brain pathologies related to the onset of overt psychosis. Interestingly, clinical high-risk individuals for psychosis (20, 46, 47), but not genetic high-risk subjects (48–50), likely exhibit gray matter reduction of the insular cortex especially for those who later develop psychosis. However, as far as we know, no MRI studies to date have specifically examined the insular gross anatomy in these high-risk groups. Thus, future studies will be warranted to examine whether the insular morphology is associated with vulnerability or genetic liability to psychosis and later psychosis onset.

The present study replicated our previous findings (7) in an expanded schizophrenia sample in which patients had an altered gyral organization with well-developed insular gyri (AG, MSG, and PLG), and also revealed no significant differences in the gross anatomical features of the insular cortex between the first-episode and chronically medicated subgroups. Previous gyrification studies in schizophrenia have demonstrated both hyper- and hypo-gyrification depending on the illness stages and brain regions (18); the patients likely have hyper-gyrification of diverse cortical regions in early stages (15, 51) but exhibit a progressive decline in brain gyrification predominantly in the fronto-temporal regions during the course of the illness (52). On the other hand, the present results appear to support insular gross anatomical features representing a stable neurodevelopmental marker regardless of illness stages. However, their contribution to clinical characteristics differed with the illness stage, with a developed AG being associated with an early illness onset, which implies prominent early developmental abnormalities (53), and severe positive symptoms specifically in the first-episode subgroup. We also demonstrated that a higher number of left short gyri was associated with a higher medication dose specifically in first-episode schizophrenia patients, supporting that gross anatomical features of the insular cortex may contribute to severe symptomatology that requires higher dose of medication at early illness stages. Interestingly, previous studies on first-episode schizophrenia also supported hyper-gyrification (15) and dysfunctional connectivity (54) in the anterior insular subdivision being associated with the severity of positive symptoms, implicating the contribution of early developmental processes associated with gyral formation in the anterior insula to the later production of psychotic symptoms. On the other hand, the relationships between the insular gross anatomy, a stable brain feature, and clinical features in chronic patients need to be interpreted with caution because the latter may be affected by a number of factors (e.g., medication and the chronicity of illness). However, it is possible that the insular gross morphology also contributes to the clinical course (e.g., treatment response) and stable clinical characteristics associated with specific subtypes.

Indeed, the present results suggest that the insular gross anatomy is associated with a persistent trait-like clinical feature of schizophrenia. No significant differences were observed in the number of insular gyri between the deficit and non-deficit subtypes of schizophrenia; however, the deficit subgroup was characterized by a more well-developed right MSG than the non-deficit subgroup. While etiological factors related to deficit schizophrenia have yet to be identified, the relationships between deficit schizophrenia and premorbid maladjustment (25, 55), general cognitive impairments (56), and neurological anomalies (27) appear to support pervasive abnormalities in neurodevelopment in this specific subtype (26, 27). The present results are consistent with previous MRI findings showing enhanced interregional cortical coupling, which may reflect reduced network differentiation during early neurodevelopment (39), and alterations in the gross brain morphology (e.g., gyrification patterns) (28, 30) specifically in deficit schizophrenia. Although the exact role of the MSG in the human brain remains unclear, the present result showing its relationship with persistent negative symptoms in schizophrenia supports functional neuroimaging evidence showing the crucial involvement of the regional functional organization within the insular cortex of the short insula (incl. the MSG) in social-emotional networks, particularly on the right hemisphere (1, 3, 57).

There are several potential limitations in this study that need to be addressed. Although the insular cortex has a number of functions in a range of cognitive domains (57) that are impaired in schizophrenia (e.g., emotional, auditory processing, and language-related functions) (58), the present study did not systematically assess cognition in participants. Therefore, it remains unclear whether the insular gross anatomy in schizophrenia spectrum disorders is associated with cognitive impairments. Furthermore, since insular gross anatomical diversity itself is widely observed in healthy subjects, its relationship with brain function warrants further study, for example, using functional/connectivity neuroimaging. Another limitation in the present study is that the deficit and non-deficit schizophrenia subgroups were not matched for sex, potentially reflecting the general tendency that male sex is associated with deficit schizophrenia (59). Since male schizophrenia patients had a higher prevalence of a developed right MSG than female patients, our results on the schizophrenia subtype need to be replicated in a larger and/or more sex-balanced cohort. Moreover, schizophrenia patients were older than healthy controls. However, the present results did not change even when we statistically controlled for the age difference. In addition, because altered brain gyrification is also observed in other neuropsychiatric disorders, such as bipolar disorder [reviewed by Sasabayashi et al. (18)], the disease specificity of our gross insular findings in schizophrenia spectrum disorders needs to be examined in further studies.

In summary, the present MRI study on gross anatomical features in the insular cortex support schizophrenia and SPD patients having similar brain characteristics possibly on the basis of common vulnerability associated with early neurodevelopmental anomalies. In schizophrenia, the insular gross anatomy appears to be associated with symptom severity, particularly in the early illness stages, as well as persistent traits associated with the deficit syndrome. However, the functional significance of this gross anatomical variation needs to be investigated in more detail in patients with neuropsychiatric disorders and healthy controls.

## Data availability statement

The raw data supporting the conclusions of this article will be made available by the authors, without undue reservation.

## Ethics statement

The studies involving human participants were reviewed and approved by the Committee of Medical Ethics of the University of Toyama. Written informed consent to participate in this study was provided by the participants’ legal guardian/next of kin.

## Author contributions

MS and TT conceived the idea and methodology of the study. TT conducted the statistical analyses and wrote the manuscript. DS, YT, and HK recruited subjects and were involved in clinical and diagnostic assessments. TT and DS analyzed the MRI data. KN provided technical support for MRI scanning and data processing. AF and YY managed the MRI and clinical data. MS and YT contributed to the writing and editing of the manuscript. All authors contributed to the article and approved the submitted version.
