# Supplementary material for: Gross anatomical features of the insular cortex in schizophrenia and schizotypal personality disorder: Potential relationships with vulnerability, illness stages, and clinical subtypes
Source: Front Psychiatry. 2022 Nov 18;13:1050712. doi: 10.3389/fpsyt.2022.1050712 (PMC9715601; doi:10.3389/fpsyt.2022.1050712)
Supplement: Supplementary file 1 [file Table_1.docx]

sTable 1. Sample characteristics and gross insular morphology of participants (except for first-episode schizophrenia).

	Controls (*N* = 88)	SPD (*N* = 47)	Sz (*N* = 62)	Group differences
M/F	49/39	29/18	27/35	Chi-square = 3.90, p = 0.142
Age (years)	24.1 ± 6.0	25.0 ± 5.4	29.6 ± 6.2	F (2, 194) = 16.90, p < 0.001; Controls < Sz
Handedness (right/left/mixed)	88/0/0	47/0/0	57/1/4	Fisher's exact test, p = 0.008
Height (cm)	166.3 ± 7.8	165.9 ± 8.7	164.1 ± 8.1	F (2, 194) = 1.35, p = 0.262
Education (years)	15.7 ± 3.0	13.1 ± 2.0	13.6 ± 2.2	F (2, 194) = 20.52, p < 0.001; SPD, Sz < Controls
Parental education (years)[a]	13.0 ± 2.3	12.3 ± 1.7	11.9 ± 1.9	F (2, 185) = 4.41, p = 0.013; Sz < Controls
Onset age (years)	-	-	21.8 ± 5.4	-
Duration of Illness (years)	-	-	7.6 ± 4.1	-
Medication dose (HPD equivalent, mg/day)	-	4.8 ± 5.7	9.6 ± 8.3	F (1, 107) = 11.83, p < 0.001; SPD < Sz
Duration of medication (years)	-	1.5 ± 3.0	5.3 ± 4.3	F (1, 107) = 25.87, p < 0.001; SPD < Sz
Medication type (atypical/typical/mixed)	-	26/14/0	28/27/5	Fisher's exact test, p = 0.073
Total SAPS scores[b]	-	16.0 ± 9.2	31.8 ± 22.6	F (1, 100) = 19.40, p < 0.001; SPD < Sz
Total SANS scores[b]	-	41.9 ± 21.7	49.0 ± 19.6	F (1, 100) = 3.03, p = 0.085
Number of short gyri				F (2, 191) = 28.22, p < 0.001; Controls < SPD, Sz
Left	2.78 ± 0.58	3.32 ± 0.66	3.26 ± 0.77	
Right	2.70 ± 0.59	3.36 ± 0.67	3.11 ± 0.60	
Number of long gyri				F (2, 191) = 9.07, p < 0.001; Controls < SPD, Sz in M
Left	1.85 ± 0.42	2.04 ± 0.20	2.05 ± 0.42	
Right	1.85 ± 0.44	1.98 ± 0.33	2.05 ± 0.38	
AG (absent/underdeveloped/developed)				
Left	36/33/19	17/16/14	21/13/28	Chi-square = 10.35, p = 0.035
Right	44/32/12	13/15/19	27/17/18	Chi-square = 13.97, p = 0.007
MSG (absent/underdeveloped/developed)				
Left	16/25/47	1/3/43	6/8/48	Chi-square = 23.68, p < 0.001
Right	11/31/46	2/3/42	4/7/51	Chi-square = 26.83, p < 0.001
PLG (absent/present)				
Left	15/73	0/47	4/58	Chi-square = 11.27, p = 0.004
Right	16/72	3/44	5/57	Chi-square = 5.42, p = 0.067

Values represent means ± SDs unless otherwise stated.

AG, accessory gyrus; ALG, anterior long gyrus; F, female; HPD, haloperidol; M, male; MSG, middle short gyrus; PLG, posterior long gyrus; SANS, scale for the assessment of negative symptoms; SAPS, scale for the assessment of positive symptoms; SPD, schizotypal personality disorder; Sz, schizophrenia.

[illegible]
